# Persistent eosinopenia is associated with in-hospital mortality among older patients: unexpected prognostic value of a revisited biomarker

**DOI:** 10.1186/s12877-021-02515-0

**Published:** 2021-10-14

**Authors:** Bethsabee Partouche, Marion Pepin, Pauline Mary de Farcy, Jean-Emmanuel Kahn, Bruno Sawczynski, Laurent Lechowski, Laurent Teillet, Frederic Barbot, Marie Herr, Benjamin Davido

**Affiliations:** 1 Geriatrics Department, Paris-Saclay University, Versailles Saint Quentin en Yvelines University (UVSQ), AP-HP Ambroise Paré Hospital, 92100 Boulogne-Billancourt, France; 2 Paris-Saclay University, UVSQ, Inserm, CESP, Clinical Epidemiology, 92100 Boulogne Billancourt, France; 3 Geriatrics Department, Paris-Saclay University, UVSQ, AP-HP, Ste Périne Hospital, 75016 Paris, France; 4 Internal Medicine Department, Paris-Saclay University, UVSQ, AP-HP, Ambroise Paré Hospital, 92100 Boulogne-Billancourt, France; 5 Medical Information Department (DIM), Paris-Saclay University, AP-HP, Ambroise Paré Hospital, 92100 Boulogne-Billancourt, France; 6 Paris-Saclay University, AP-HP, Raymond Poincaré Hospital, Clinical Investigation Center, Inserm (CIC 1429), 92380 Garches, France; 7 Epidemiology and Public Health Department, Paris-Saclay University, UVSQ, Inserm, CESP, Anti-infective evasion and pharmacoepidemiology; AP-HP, Raymond-Poincaré Hospital, 92380 Garches, France; 8 Infectious and Tropical Disease Department, Paris-Saclay University, UVSQ, AP-HP, Raymond Poincaré Hospital, 92380 Garches, France

**Keywords:** Eosinopenia, Geriatrics, Bacterial infection, Outcome, Mortality

## Abstract

**Introduction:**

Infection is one of the major causes of mortality and morbidity in older adults. Available biomarkers are not associated with prognosis in older patients. This study aimed to analyze the value of eosinopenia (eosinophil count< 100/mm^3^) as a prognosis marker among older patients with suspected or confirmed bacterial infection.

**Methods:**

A retrospective study was performed from 1 January to 31 December 2018 among patients in a geriatrics ward suffering from a bacterial infection treated with antibiotics. Biomarker data including the eosinophil count, neutrophil count and C-reactive protein (CRP) were collected within 4 days after patient diagnosis. Persistent eosinopenia was defined as a consistent eosinophil count< 100/mm^3^ between Day 2 and Day 4. The association of biomarkers with 30-day hospital mortality in a multivariate analysis was assessed and their predictive ability using the area under the ROC curve (AUC) was compared.

**Results:**

Our study included 197 patients with a mean age of 90 ± 6 years. A total of 36 patients (18%) died during their stay in hospital. The patients who died were more likely to have persistent eosinopenia in comparison to survivors (78% versus 34%, *p* < 0.001). In the multivariate analysis, persistent eosinopenia was associated with in-hospital mortality with an adjusted HR of 8.90 (95%CI 3.46–22.9). The AUC for eosinophil count, CRP and neutrophil count between Day 2 and Day 4 were 0.7650, 0.7130, and 0.698, respectively.

**Conclusion:**

Persistent eosinopenia within 4 days of diagnosis of bacterial infection appeared to be a predictor of in-hospital mortality in older patients.

**Supplementary Information:**

The online version contains supplementary material available at 10.1186/s12877-021-02515-0.

## Key points

- Eosinophil count is a cost-effective revisited biological marker
- Eosinopenia seems to be a valuable prognostic marker among older patients
- Such marker during bacterial infection can be useful to predict in-hospital mortality

## Introduction

Infection is one of the major causes of mortality and morbidity in older patients [1]. Between 2000 and 2009, mortality associated with infection accounted for 5% of all deaths worldwide. Currently used biological markers (biomarkers) such as C-reactive protein (CRP) and procalcitonin (PCT) lack discriminatory power to diagnose infection in older patients (> 65 years) [2, 3]. Regarding prognostic approach, several biomarkers were evaluated on the day before treatment initiation to predict mortality among critically ill patients suffering from bacterial infection in an intensive care unit (ICU). Jensen et al. evaluated CRP value> 9 mg/L, leukocytes> 10 G/L and PCT > 1 ng/mL and showed that the only relevant parameter to identify patients at increased risk of all-cause mortality on day 90 was PCT in adults (median age 57 years) [4]. However, PCT has shown deviations with imperfect sensitivity and specificity in older adults [2, 5, 6]. Clinical need for reliable biomarkers in older patients is particularly important as they may present without classical signs and symptoms of infection [2].

In common practice, evaluation of inflammation encountered during a bacterial infection is provided by a white blood cell (WBC) count. It is cost-effective and used in medicine or surgery wards as well as in primary care to support clinical findings which, when abnormal, provoke further investigation. Polymorphonuclear neutrophils (PMN) are usually increased in bacterial infection or inflammation cases whereas lymphopenia is suggestive of a viral infection in the general population. Eosinophils are WBCs that normally account for 1 to 3% of total leukocytes [7]. Eosinopenia is defined by a reduced eosinophil count (EC) and was considered as a possible marker of infection by Zappert et al. in early 1893 [8]. In 1929, Schilling et al. suggested that eosinopenia was related to bacterial infection [9], whereas at a later date, Bass et al. suggested that its pathophysiology was related to the migration of eosinophils into the inflammatory site during the acute phase of inflammation [10]. To date, only a few studies confirm this hypothesis, however, there is no consensual definition of eosinopenia without a defined threshold of values ranging from 10 to 140 eosinophils/mm^3^ [11–13].

Moreover, eosinopenia at hospital admission has been reported to be associated with a worse prognosis. Abidi et al. showed that in ICU, the deeper the eosinopenia, the poorer the prognosis [14]. Two other studies have shown that eosinopenia was a marker of mortality in different populations (at the ICU and respiratory medicine ward but not in geriatrics) [14, 15]. Similarly, a recent study found that eosinophil count at admission was strongly associated with mortality risk in patients suffering from *Clostridium difficile* infection [16]. EC is a dynamic parameter, it tends to normalize rapidly after an effective antimicrobial therapy [11]. A few studies described that normalization of eosinopenia could be a predictive marker of in-hospital favorable evolution [12, 17, 18].

To the best of our knowledge, a study evaluating the prognostic role of persistent eosinopenia during infection among geriatric patients does not exist. The aim of this study was to analyze the value of eosinopenia as a prognostic marker in such patients.

## Methods

### Study design and population

An observational, retrospective single-center study was performed in a teaching hospital in Paris, France (*Ambroise Paré Hospital in Boulogne-Billancourt*). The hospital’s information system, which is routinely managed by healthcare staff for the financing of hospital activities (*Programme de Médicalisation des Systèmes d’Information* – PMSI), was used to identify eligible patients. Patients who were hospitalized in acute geriatric wards between 1 January and 31 December 2018 with a diagnosis of bacterial infection requiring initiation of an antimicrobial therapy were included. Diagnosis of presumed bacterial infection was made by the clinician in charge of the patient. Patients that received antimicrobial therapy, without alternative diagnosis and with a diagnosis of bacterial infection confirmed in hospital medical report were included. Suspected or confirmed bacterial infections were respiratory, urinary, digestive, cutaneous, cardiac and the central nervous system as well as those with bacteremia (according to ICD-10 codes that are available on request). Data about bacterial infections were subsequently retrieved from patient medical records. Of note, since November 2012, a remote infectious disease specialist consultant, working part-time has been specifically devoted to promote antibiotic stewardship and advices to all hospital departments on demand. The consultant also performs post-prescription antibiotic review using computerized tools with shared access, and e-mail alerts are generated on day 3 by the pharmacist, leading to a revaluation of broad-spectrum antibiotics.

Exclusion criteria were bone and joint infections because of specific aspects in the management of these infections which require surgical procedures as well as factors that could modify the course of the infection and EC interpretation such as immunosuppressive treatments (for example corticosteroid therapy at a dose≥10 mg/d prednisone equivalent, anti-cancer chemotherapy and/or methotrexate), hematological malignancies. Patients already under an antimicrobial regimen for more than 48 h prior to admission were also excluded. In cases of patients with multiple stays over the study period, only the last stay was included in the analysis.

### Data collection

Data were collected from patient medical records using Agfa® Orbis software. Our main outcome was in-hospital mortality. The variable was coded “1” if the patient died during hospital stay within 30 days of diagnosis and “0” if they were still hospitalized after 30 days and/or discharged before the endpoint. Data about biomarkers including EC, PMN and CRP were collected during the week following the diagnosis of a bacterial infection defined as Day 0 (D0). The following 4 days after D0 were defined as Day 1 (D1), Day 2 (D2), Day 3 (D3) and Day 4 (D4).

As a retrospective study in routine care, the frequency of biological monitoring varied depending on the patient. To summarize the information available about the evolution of EC after D0, a variable capturing the maximum value of EC between D2 and D4 considering the minimum value of PMN and CRP at these time points was created. Eosinopenia was defined by EC < 100 eosinophils/mm^3^ (0.1 G/L), severely elevated CRP by a value> 100 mg/L (consistent with previous studies) [19] and elevated PMN by a value> 7000/mm^3^ in accordance with established professional agreements [20].

Other variables included in the analysis were demographics (age and sex), comorbidities (Charlson score: weight severity of 19 different comorbidities, highest score indicating severity) [21], malnutrition (moderate: albuminemia< 35 g/L or BMI < 21 kg/m^2^; severe: albuminemia< 30 g/L or BMI < 18 kg/m^2^), bedsore, estimated glomerular filtration rate (eGFR calculated with CKD-EPI), characteristics of the infection (type of infection, initiation of antibiotics), sepsis assessed by the recent bedside clinical score termed quickSOFA completed by a SOFA score if needed according to the definition of “SEPSIS-3” and ICU stay [22].

### Statistical analysis

Descriptive statistics used mean ± standard deviation (SD) for normally distributed continuous variables and median and interquartile range for non-normally distributed variables including eosinophil count. Numbers and percentages were used to describe categorical variables. Factors associated with in-hospital mortality were investigated among demographics, clinical and biological variables using the unequal variance Student’s test or the Wilcoxon Signed-Rank test for continuous variables and the Chi^2^ test or Fischer’s exact test for categorical variables. The survival curves as a function of eosinopenia from D0 to between D2 and D4 were carried out according to the Kaplan-Meier method. Multivariate analysis was performed using a Cox proportional hazards model. Variables that were introduced into the model included age, gender and all variables associated with a *p* < 0.20 in bivariate analysis. Variables associated with a *p* > 0.10 were excluded from the model in a second step. Similar models were run for PMN and CRP. Results are expressed as Hazard Ratios (HR) with 95% confidence interval (CI).

The prognostic role of EC between D2 and D4 was described in terms of sensitivity, specificity, Predictive Positive Value (PPV) and Negative Predictive Value (NPV) and was compared to the predictive ability of PMN and CRP between D2 and D4 by using the area under the Receiver Operating Characteristics (ROC) curve. Statistical analyzes were performed using Stata Software© version 15.

## Results

A total of 235 stays were eligible in this study and 197 patients were selected after the exclusion criteria. The mean age of patients was 90 ± 6 years with a sex ratio of almost 1 (Table 1). The two most common infections (89% of all cases) were respiratory and/or urinary tract infections. Baseline analysis observed eosinopenia in 155 patients (79%) (Table 2). The median value of EC was 10 eosinophils/mm^3^ (IQR0 to 80). A total of 36 patients died in hospital during the 30 days after admission for infection (18%). The median time to death after admission was 6 days (IQR5 to 13).

**Table 1:** Characteristics of the study sample

Variable	Total (***n*** = 197)	Patients who died in hospital within 30 days of diagnosis (***n*** = 36)	Patients discharged or alive at Day 30^a^ (***n*** = 161)	***P***-value^b^
**Age, mean ± SD**	89.6 ± 5.7	90.9 ± 6.7	89.4 ± 5.4	0.204
**Age in terciles, n (%)**				0.116
*62 to 86 years old*	66 (33.5)	7 (19.4)	59 (36.7)	
*87 to 92 years old*	74 (37.6)	15 (41.7)	59 (36.7)	
*93 to 104 years old*	57 (28.9)	14 (38.9)	43 (26.7)	
**Sex, n (%)**				0.441
*Women*	99 (50.3)	16 (44.4)	83 (51.6)	
*Men*	98 (49.8)	20 (55.6)	78 (48.5)	
**Charlson, mean ± SD**	2.9 ± 2.2	3.3 ± 2.6	2.8 ± 2.2	0.330
**Length of stay, mean ± SD** **Malnutrition, n (%)**	11.4 +/− 7.2	8.3 +/− 5.2	12.1 +/− 7.4	< 0.001 0.145
*No*	36 (18.3)	4 (11.1)	32 (19.9)	
*Moderate*	70 (35.5)	10 (27.8)	60 (37.3)	
*Severe*	91 (46.2)	22 (61.1)	69 (42.9)	
**Bedsore, n (%)**	21 (10.8)	6 (17.7)	15 (9.3)	0.218
**Type of infection, n (%)**				0.036
*Pulmonary*	112 (56.9)	26 (72.2)	86 (53.4)	
*Urinary*	43 (31.8)	2 (5.6)	41 (25.5)	
*Bacteraemia*	27 (13.7)	5 (13.9)	22 (13.7)	
*Other or combined*	15 (7.6)	3 (8.3)	12 (7.5)	
**Initiation of antibiotics, n (%)**				0.241
*Within 24 h*	111 (56.4)	25 (69.4)	86 (53.4)	
*Between 24 and 48 h*	60 (30.5)	8 (22.2)	52 (32.3)	
*After 48 h*	26 (13.2)	3 (8.3)	23 (14.3)	
**Sepsis, n (%)**	30 (15.2)	6 (16.7)	24 (14.9)	0.799
**Stay in the ICU, n (%)**	13 (6.6)	2 (5.6)	11 (6.8)	1.000
**GFR, n (%)**				< 0.001
*≥ 60 ml/min*	126 (64.0)	16 (44.4)	110 (68.3)	
*≥ 30 and < 60 ml/min*	48 (24.4)	9 (25.0)	39 (24.2)	
*< 30 ml/min*	23 (11.7)	11 (30.6)	12 (7.5)	

*BMI* Body Mass Index, *GFR* Glomerular Filtration Rate according to CKD-EPI, *ICU* Intensive care unit, *SD* Standard Deviation

^a^156 patients discharged and 5 still hospitalized at Day 30

^b^Chi2 test or Fischer exact test for comparisons of proportions and Student t-test with unequal variance for comparisons of means

**Table 2:** Eosinophil count, neutrophil count and C-reactive protein (CRP) on the date of diagnosis of bacterial infection (D0) and between Day 2 and Day 4 (D2-D4) according to in-hospital mortality

Variable	Total (***n*** = 197)	Patients who died in hospital within 30 days of diagnosis (***n*** = 36)	Patients discharged or alive at Day 30^a^ (***n*** = 161)	***P***-value^b^
**Eosinophil count (EC)**				
**D0**				
*Continuous, median (IQR)*	10 (0 to 80)	10 (0 to 45)	20 (0 to 90)	0.058
*EC < 100 /mm*^*3*^*, n (%)*	155 (78.7)	32 (88.9)	123 (76.4)	0.098
**D2-D4 (highest value)**				
*Continuous, median (IQR)*	130 (30 to 230)	25 (5 to 80)	140 (60 to 250)	< 0.001
*EC < 100 /mm*^*3*^*, n (%)*	82 (41.6)	38 (77.8)	54 (33.5)	< 0.001
**Neutrophil count (NC)**				
**D0**				
*Continuous, median (IQR)*	10,910 (7735 to 16,400)	12,100 (8980 to 18,020)	10,590 (7590 to 15,150)	0.126
*NC > 7000/mm*^*3*^*, n (%)*	159 (81.1)	30 (85.7)	129 (80.1)	0.634
**D2-D4 (lowest value)**				
*Continuous, median (IQR)*	6900 (4970 to 10,270)	9660 (7090 to 13,475)	6600 (4730 to 8750)	< 0.001
*NC > 7000/mm*^*3*^*, n (%)*	97 (49.2)	28 (77.8)	69 (42.9)	< 0.001
**CRP**				
**D0**				
*Continuous, median (IQR)*	91 (39 to 180)	147 (97 to 189)	80 (35 to 169)	0.014
*CRP > 100 mg/L, n (%)*	80 (45.5)	22 (71.0)	58 (40.0)	0.003
**D2-D4 (lowest value)**				
*Continuous, median (IQR)*	103 (50 to 170)	177 (98 to 208)	95 (47 to 159)	< 0.001
*CRP > 100 mg/L, n (%)*	94 (50.5)	25 (73.5)	69 (45.4)	0.004

^a^Five patients still hospitalized at Day 30 and 156 patients discharged before

^b^Wilcoxon rank test

The comparison of baseline characteristics of patients according to in-hospital mortality revealed that the patients who died were more likely to have suffered from a respiratory infection, had a lower eGFR using the CKD-EPI formula and had a higher value of CRP compared to surviving patients (Table 1). Whereas eosinopenia at baseline did not differ between groups, eosinopenia between D2 and D4 was more frequent in patients who died compared to survivors (78% versus 34% among the survivors, *p* < 0.001) (Table 2).

**Fig. 1:**
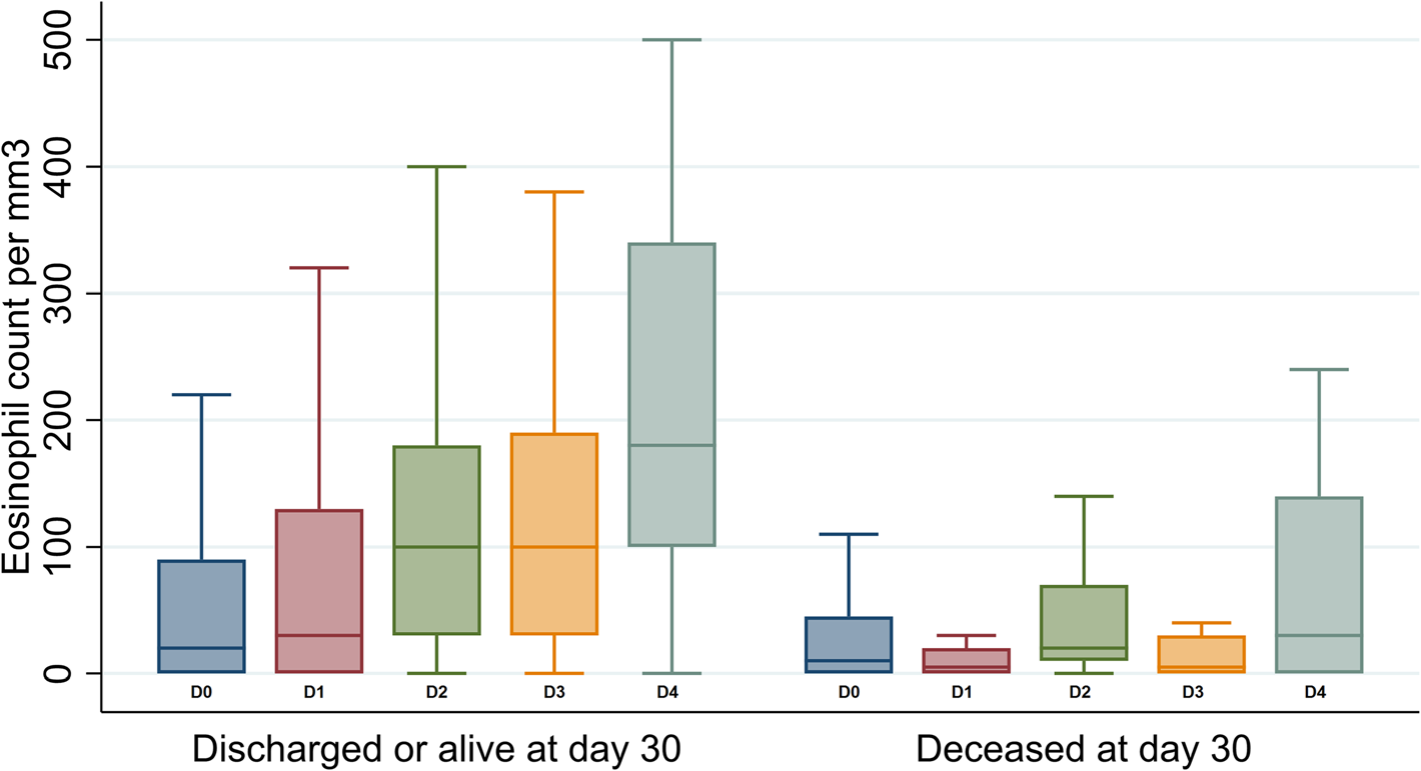
Eosinophil count within five days of diagnosis of a bacterial infection according to in-hospital mortality. Number of patients with eosinophil count: Discharged or alive at Day 30, Day 0: *n* = 161, Day 1: *n* = 63, Day 2: *n* = 97, Day 3: *n* = 61, Day 4: *n* = 70. Deceased at day 30: *n* = 36 on day 0, *n* = 12 on day 1, *n* = 30 on day 2, *n* = 8 on day 3, *n* = 18 on day 4

**Fig. 2:**
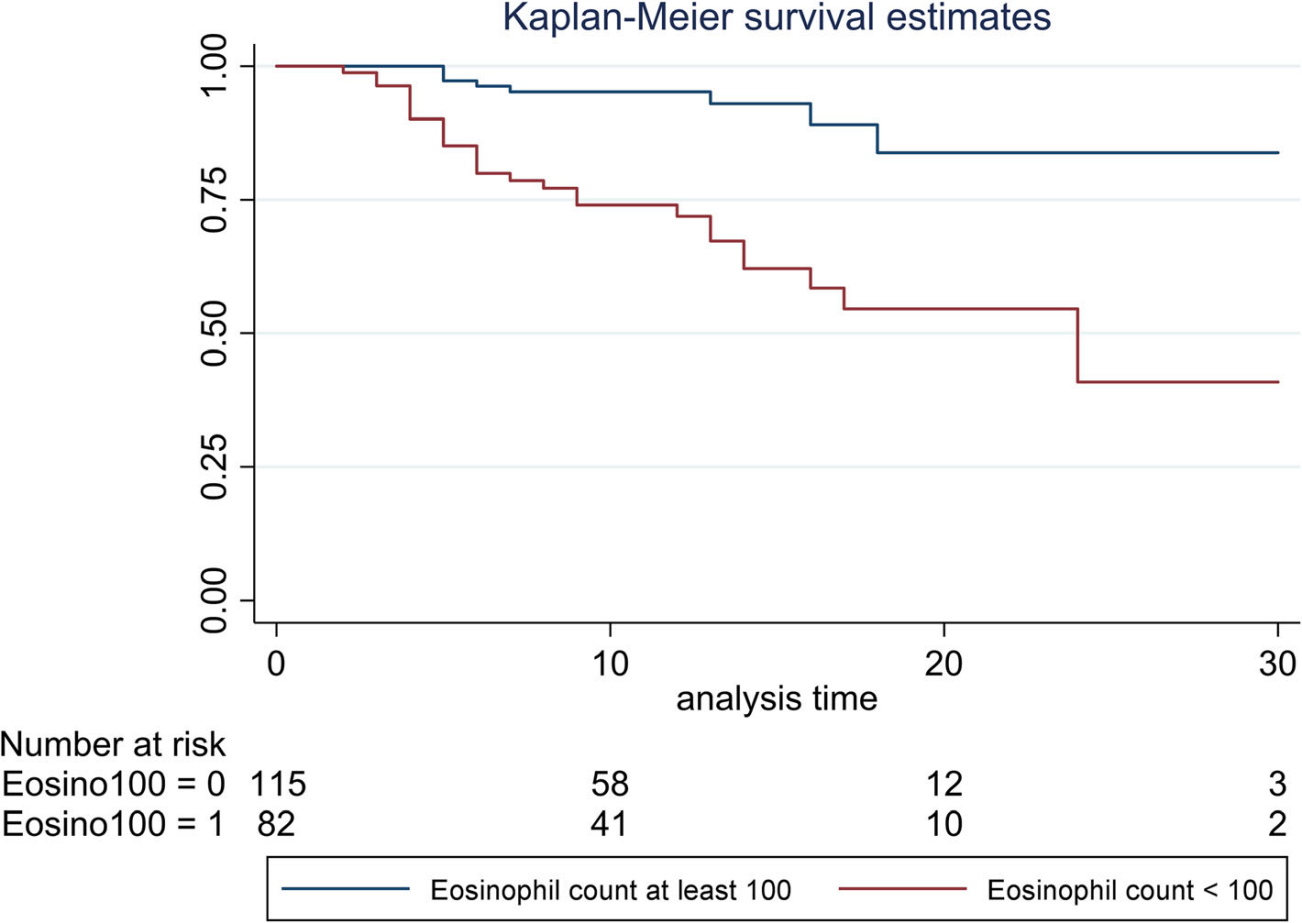
Survival curves according to maximum eosinophil between Day 2 and Day 4 among patients with a diagnosis of infection (*N* = 197)

Detailed information on the evolution of EC within the 4 days of diagnosis and survival curves according to eosinopenia on or between D2-D4 are shown in Figs. 1 and 2 respectively. Clearly, patients with an EC < 100/mm^3^ had poorer prognosis than those with EC above 100/mm^3^ (*p* < 0.001). After adjustment for any confounders, EC below 100/mm^3^ between D2 and D4 was an independent predictor of in-hospital mortality with an adjusted HR of 8.90 (95% CI 3.46 to 22.9) (Table 3). A neutrophil count higher than 7000/mm^3^ between D2 and D4 was also associated with in-hospital mortality with an adjusted HR of 4.34 (95% CI 1.81 to 10.4), unlike CRP. Other factors associated with in-hospital mortality were age, respiratory tract infection and stage 4 chronic kidney disease (eGFR< 30 mL/min). A full model is presented in Supplementary Table 1.

**Table 3:** Association of eosinophil count, neutrophil count and C-reactive protein (CRP) between Day 2 and Day 4 with in-hospital mortality within 30 days of diagnosis of infection

Variable	Deceased at day 30, n (%)	Crude HR (95%CI%)	Adjusted HR* (95%CI)	***P***-value
**Eosinophil count < 100/mm**^**3**^				
*No*	8 (7.0)	1	1	
*Yes*	28 (34.2)	5.00 (2.28 to 11.0)	8.90 (3.46 to 22.9)	< 0.001
**Neutrophil count > 7000/mm**^**3**^				
*No*	8 (8.00)	1	1	
*Yes*	28 (28.9)	3.48 (1.58 to 7.64)	4.34 (1.81 to 10.4)	0.001
**CRP > 100 mg/L**				
*No*	9 (9.8)	1	1	
*Yes*	25 (26.6)	2.33 (1.08 to 5.03)	1.65 (0.74 to 3.68)	0.222

Multivariate Cox model adjusted for sex, age, Charlson comorbidity index, undernutrition, type of infection and glomerular filtration rate

The AUC for EC, CRP and PMN between D2 and D4 were 0.7650, 0.7130 and 0.698, respectively (Supplementary Figures 1, 2 and 3). Overall, EC had the best values of sensitivity (77.8%), specificity (66.5%), PPV (34.2%), and PPN (93.0%) of these three biomarkers (data for PMN and CRP is presented in Supplementary Table 2).

## Discussion

Our study showed that persistent eosinopenia within 4 days after the initial diagnosis of bacterial infection was strongly associated with 30-day in-hospital mortality in older patients. Furthermore, the differentiating power of eosinopenia between D2 and D4 suggests its potential value as a prognostic marker of in-hospital mortality.

The favorable evolution of EC following admission has already been addressed in a study by Terradas et al. [17] in which normalization of EC after D3 was observed amongst surviving patients. This finding supports our results even though our patients were notably older. In contrast, better assessing patient’s clinical course when EC does not increase as expected should alert the physician to reevaluate the antimicrobial therapy in light of microbiological findings and antimicrobial susceptibility testing. This is particularly relevant in the daily practice of geriatricians where the early and appropriate management of bacterial infections is crucial in preventing the exacerbation of pre-existing conditions that are often responsible for prolonged hospitalization and loss of autonomy. In addition, the association of prolonged antimicrobial therapy with a differential outcome than a shorter duration of antibiotic treatment was not supported in literature [23]. As an example, shortened antibiotic duration (3 days) in patients (including older patients) with stability criteria during acute pneumonia was non-inferior to 8 days of treatment [24]. It could be insightful to evaluate the interest of EC in future studies to guide the duration of antimicrobial therapy and discontinue it as soon as a patient recovers from eosinopenia.

In literature, admission eosinopenia was a noteworthy early marker of mortality during hospitalization in different settings, namely critically-ill infected patients in ICU and patients admitted in a respiratory medicine ward for exacerbation of chronic obstructive pulmonary disease (COPD) [14, 15]. However, baseline eosinopenia was frequent (almost 80%) and less relevant in predicting in-hospital mortality in our geriatric population. Interestingly, in another study by Akagi et al., PCT was not an independent predictor of mortality from respiratory tract infections in older patients unlike persistent eosinopenia as suggested in our findings [25].

In addition, our multivariate analyses confirmed that respiratory tract infections were associated with in-hospital mortality as previously described and expected [26]. Similarly, as EC becomes normalized over time, CRP kinetic decreases under antimicrobial therapy in community-acquired bloodstream infection which is predictive of short or long-term mortality in a relatively young population (mean age 66.7 years) [27]. To date, there is no reliable biomarker to predict the favorable evolution during bacterial infection in the long run, however, a 2-fold decrease in the CRP level or an 80% reduction in the PCT value during follow-up is recognized as a strong argument [28]. Nevertheless, these biomarkers may come at a price that is not always affordable in low-income countries as opposed to EC analysis. Indeed, EC could help postpone a switch in antimicrobial therapy when the CRP level increases during hospitalization caused by inflammation that is other than infection.

Our study had some limitations. Firstly, it was an observational, retrospective and single-center study. As a result, the relevance of the prescribed antimicrobial therapies could not be discussed. Indeed, our study was not designed to evaluate the relevance of the chosen antimicrobial therapy as to its indication, diffusion and duration which might have played a role in the outcome. However, this did not interfere with the biological interpretation of the EC. In addition, the community-acquired or hospital-acquired nature of the infection was not mentioned in the medical chart. Secondly, our sample size was modest (*n* = 197) and can be criticized based on a lack of statistical power even though the association between persistent eosinopenia and in-hospital mortality was found significant.

Regarding strengths, our main outcome, namely in-hospital mortality within 30 days, was an objective criterion. Secondly, this study considered geriatric patients who are less often included in clinical research [29]. To our knowledge, value in biomarkers during hospitalization as a prognosis marker of any bacterial infection has rarely been evaluated in geriatric populations. Nevertheless, it should be kept in mind in the context of the COVID-19 pandemic that eosinopenia has not been established as a method to distinguish viral infection from bacterial infection in adults.

In fact, it was emphasized by Debray et al. concerning meningitis in pediatrics that the severity of an infection, rather than its bacterial characteristic, was associated with eosinopenia [12]. Moreover, it was reaffirmed that EC should be interpreted with caution in cases of COVID-19 because it can mimic a true bacterial infection as is the case with other biomarkers [30].

## Conclusion

In conclusion, our study found that persistent eosinopenia below 100/mm^3^ within 4 days of the diagnosis of bacterial infection appears to be a predictor of in-hospital mortality in older patients. Further research is needed to investigate whether the evolution of EC could help guide antimicrobial therapy duration physician decisions. Eosinopenia is easy to detect with a simple WBC count in any patient suspected of bacterial infection with no additional processing cost.

## Supplementary Information

**Additional file 1: Supplementary Table 1.** Multivariate analysis of the factors associated with in-hospital mortality within 30 days of diagnosis of an infection. **Supplementary Table 2.** Performance of eosinophil count, C-reactive protein (CRP) and neutrophil count between Day 2 and Day 4 to predict in-hospital mortality within 30 days of diagnosis of a bacterial infection. **Supplementary Figure 1.** ROC curve for in-hospital mortality within 30 days of diagnosis of bacterial infection according to the eosinophil count> 100/mm^3^ between Day 2 and Day 4. The classification variable was inverted in this analysis because higher eosinophil count is expected to predict survival. **Supplementary Figure 2.** ROC curve for in-hospital mortality within 30 days of diagnosis of a bacterial infection according to the neutrophil count> 7000/mm^3^ between Day 2 and Day 4. **Supplementary Figure 3.** ROC curve for in-hospital mortality within 30 days of diagnosis of a bacterial infection according to the C-reactive protein (CRP) > 100 mg/l between Day 2 and Day 4.

## Data Availability

The datasets used and/or analyzed during the current study are available from the corresponding author on reasonable request.

## References

[1] Gavazzi G, Krause KH (2002). Ageing and infection. Lancet Infect Dis.

[2] van Duin D (2012). Diagnostic challenges and opportunities in older adults with infectious diseases. Clin Infect Dis Off Publ Infect Dis Soc Am.

[3] Prendki V, Malézieux-Picard A, Azurmendi L, Sanchez JC, Vuilleumier N, Carballo S, Roux X, Reny JL, Zekry D, Stirnemann J, Garin N, on behalf of the PneumOldCT study group (2020). Accuracy of C-reactive protein, procalcitonin, serum amyloid a and neopterin for low-dose CT-scan confirmed pneumonia in elderly patients: a prospective cohort study. PLoS One.

[4] Jensen JU, Heslet L, Jensen TH, Tvede M (2006). Procalcitonin increase in early identification of critically ill patients at high risk of mortality*. Crit Care Med.

[5] Chenevier-Gobeaux C, Trabattoni E, Elfassy Y, Picard C, Guérin S, Borderie D, Claessens YE (2012). Decisional procalcitonin thresholds are not adapted toelderly patients admitted to the emergency room. Biomarkers.

[6] Caterino JM, Scheatzle MD, Forbes ML, D’Antonio JA (2004). Bacteremic elder emergency department patients: procalcitonin and white count. Acad Emerg Med Off J Soc Acad Emerg Med.

[7] Gibot S, Kolopp-Sarda MN, Béné MC, Cravoisy A, Levy B, Faure GC, Bollaert PE (2004). Plasma level of a triggering receptor expressed on myeloid Cells-1: its diagnostic accuracy in patients with suspected Sepsis. Ann Intern Med.

[8] Zappert J. Ueber das Vorkommen der Eosinophilen Zellen in menschlichen Blute. Z Klin Med. 1893;23:227–308.

[9] Schilling V (1929). The blood picture. CV: Mosby Co.

[10] Bass DA, Gonwa TA, Szejda P, Cousart MS, DeChatelet LR, McCall CE (1980). Eosinopenia of acute infection: production of eosinopenia by chemotactic factors of acute inflammation. J Clin Invest.

[11] Davido B (2017). Changes in eosinophil count during bacterial infection: revisiting an old marker to assess the efficacy of antimicrobial therapy. Int J Infect Dis IJID Off Publ Int Soc Infect Dis.

[12] Debray A, Nathanson S, Moulin F, Salomon J, Davido B (2019). Eosinopenia as a marker of diagnosis and prognostic to distinguish bacterial from aseptic meningitis in pediatrics. Eur J Clin Microbiol Infect Dis Off Publ Eur Soc Clin Microbiol.

[13] Abidi K, Khoudri I, Belayachi J, Madani N, Zekraoui A, Zeggwagh A, Abouqal R (2008). Eosinopenia is a reliable marker of sepsis on admission to medical intensive care units. Crit Care Lond Engl.

[14] Abidi K, Belayachi J, Derras Y, Khayari ME, Dendane T, Madani N, Khoudri I, Zeggwagh AA, Abouqal R (2011). Eosinopenia, an early marker of increased mortality in critically ill medical patients. Intensive Care Med.

[15] Holland M, Alkhalil M, Chandromouli S, Janjua A, Babores M (2010). Eosinopenia as a marker of mortality and length of stay in patients admitted with exacerbations of chronic obstructive pulmonary disease. Respirol Carlton Vic.

[16] Kulaylat AS, Buonomo EL, Scully KW, Hollenbeak CS, Cook H, Petri WA, Stewart DB (2018). Development and validation of a prediction model for mortality and adverse outcomes among patients with peripheral Eosinopenia on admission for Clostridium difficile infection. JAMA Surg.

[17] Terradas R, Grau S, Blanch J, Riu M, Saballs P, Castells X, Horcajada JP, Knobel H (2012). Eosinophil count and neutrophil-lymphocyte count ratio as prognostic markers in patients with bacteremia: a retrospective cohort study. PLoS One.

[18] Karakonstantis S, Gryllou N, Papazoglou G, Lydakis C (2019). Eosinophil count (EC) as a diagnostic and prognostic marker for infection in the internal medicine department setting. Romanian J Intern Med Rev Roum Med Interne.

[19] Dupond JL, de Wazières B, Million P, Humbert P, Gibey R (1990). Neutrophilic leukocytosis of systemic or bacterial origin: discriminative C-reactive protein?. Rev Med Interne.

[20] Adeli K, Raizman JE, Chen Y, Higgins V, Nieuwesteeg M, Abdelhaleem M, Wong SL, Blais D (2015). Complex biological profile of hematologic markers across pediatric, adult, and geriatric ages: establishment of robust pediatric and adult reference intervals on the basis of the Canadian health measures survey. Clin Chem.

[21] Charlson M, Szatrowski TP, Peterson J, Gold J (1994). Validation of a combined comorbidity index. J Clin Epidemiol.

[22] Singer M, Deutschman CS, Seymour CW, Shankar-Hari M, Annane D, Bauer M, Bellomo R, Bernard GR, Chiche JD, Coopersmith CM, Hotchkiss RS, Levy MM, Marshall JC, Martin GS, Opal SM, Rubenfeld GD, van der Poll T, Vincent JL, Angus DC (2016). The third international consensus definitions for Sepsis and septic shock (Sepsis-3). JAMA.

[23] Spellberg B (2016). The new antibiotic mantra-“shorter is better”. JAMA Intern Med.

[24] Dinh A, Ropers J, Duran C, Davido B, Deconinck L, Matt M, Senard O, Lagrange A, Makhloufi S, Mellon G, de Lastours V, Bouchand F, Mathieu E, Kahn JE, Rouveix E, Grenet J, Dumoulin J, Chinet T, Pépin M, Delcey V, Diamantis S, Benhamou D, Vitrat V, Dombret MC, Renaud B, Perronne C, Claessens YE, Labarère J, Bedos JP, Aegerter P, Crémieux AC, Attal-Behar J, Beaune S, Chinet T, Cudennec T, de Laroche M, de Thezy A, Dumoulin J, DUPONT C, Fercot E, Giraut V, Greffe S, Grenet J, Guyot C, Kahn JE, Labrune S, Lachatre M, Moulias S, Naline C, Pepin M, Rouveix E, Sahut-D'izarn M, Sefssafi A, Teillet L, Bru JP, Gaillat J, Gautier V, Janssen C, Pagani L, Vitrat V, Abderrahmane M, Camuset J, Legall C, Longuet-Flandres P, Menn AM, de Lastours V, Lecronier M, Prevost G, Burdet C, Derradji O, Escaut L, Hinglais E, Lebras P, Lefevre E, Noaillon M, Rabier P, Raphael M, Teicher E, Verny C, Vittecoq D, Wyplosz B, Ben Hayoun M, Brun-Vezinet F, Casalino E, Choquet C, Dombret MC, Duval X, Houhou N, Joly V, Lescure X, Pogliaghi M, Rioux C, Yazdanpanah Y, Barros E, Begga B, Boukobza S, Bouredji H, Chouahi I, Delacroix I, Froissart A, Garrait V, Ngwem E, Phlippoteau C, Salehabadi S, Toper C, Vinas F, Amsilli M, Epaulard O, Pavese P, Pierre I, Stahl JP, Aulagnier J, Celerier J, Cojocariu R, Mathieu E, Rachline C, Schoindre Y, Sene T, Thierry C, Aparicio C, Delcey V, Lopes A, Morgand M, Sellier P, Simoneau G, Chakvetadze C, Diamantis S, Gauthier A, Jidar K, Jourdain B, Boitiaux JF, Deschamps P, Devaud E, Philippe B, Calin RO, Chroboczek T, Davido B, Deconinck l, de Truchis p, Lagrange A, Makhloufi S, Matt M, Mellon G, Senard O, Benhamou D, Chapuzet C, Chauffrey L, Etienne M, Joly LM, Obstoy B, Salaun M, Thiberville L, Tillon J, Bollens D, Bottero J, Campa P, Cosqueric G, Lefebvre B, Ouazene Z, Pacanowski J, Pateron D, Valin N, Compain C, Cordel H, Doumenc B, Fois E, Gambier N, Khuong MA, Pasqualoni e, Poupard m (2021). Discontinuing β-lactam treatment after 3 days for patients with community-acquired pneumonia in non-critical care wards (PTC): a double-blind, randomised, placebo-controlled, non-inferiority trial. Lancet Lond Engl.

[25] Akagi T, Nagata N, Miyazaki H, Harada T, Takeda S, Yoshida Y, Wada K, Fujita M, Watanabe K (2019). Procalcitonin is not an independent predictor of 30-day mortality, albeit predicts pneumonia severity in patients with pneumonia acquired outside the hospital. BMC Geriatr.

[26] Yoshikawa TT (2000). Epidemiology and unique aspects of aging and infectious diseases. Clin Infect Dis Off Publ Infect Dis Soc Am.

[27] Póvoa P (2020). C-reactive protein and albumin kinetics after antibiotic therapy in community-acquired bloodstream infection. Int J Infect Dis IJID Off Publ Int Soc Infect Dis.

[28] de Jong E, van Oers JA, Beishuizen A, Vos P, Vermeijden WJ, Haas LE, Loef BG, Dormans T, van Melsen GC, Kluiters YC, Kemperman H, van den Elsen MJ, Schouten JA, Streefkerk JO, Krabbe HG, Kieft H, Kluge GH, van Dam VC, van Pelt J, Bormans L, Otten MB, Reidinga AC, Endeman H, Twisk JW, van de Garde EMW, de Smet AMGA, Kesecioglu J, Girbes AR, Nijsten MW, de Lange DW (2016). Efficacy and safety of procalcitonin guidance in reducing the duration of antibiotic treatment in critically ill patients: a randomised, controlled, open-label trial. Lancet Infect Dis.

[29] van Deudekom FJ, Postmus I, van der Ham DJ, Pothof AB, Broekhuizen K, Blauw GJ, Mooijaart SP (2017). External validity of randomized controlled trials in older adults, a systematic review. PLoS One.

[30] Davido B, Partouche B, Jaffal K, de Truchis P, Herr M, Pepin M. Eosinopenia in COVID-19: what we missed so far? J Microbiol Immunol Infect Wei Mian Yu Gan Ran Za Zhi. 2021. 10.1016/j.jmii.2021.01.013. 10.1016/j.jmii.2021.01.013 PMC7891077 33648873

