# Supplementary material for: Persistent eosinopenia is associated with in-hospital mortality among older patients: unexpected prognostic value of a revisited biomarker
Source: BMC Geriatr. 2021 Oct 14;21:557. doi: 10.1186/s12877-021-02515-0 (PMC8516088; doi:10.1186/s12877-021-02515-0)
Supplement: Supplementary file 1 — Additional file 1: Supplementary Table 1. Multivariate analysis of the factors associated with in-hospital mortality within 30 days of diagnosis of an infection. Supplementary Table 2. Performance of eosinophil count, C-reactive protein (CRP) and neutrophil count between Day 2 and Day 4 to predict in-hospital mortality within 30 days of diagnosis of a bacterial infection. Supplementary Figure 1. ROC curve for in-hospital mortality within 30 days of diagnosis of bacterial infection according to the eosinophil count> 100/mm3 between Day 2 and Day 4. The classification variable was inverted in this analysis because higher eosinophil count is expected to predict survival. Supplementary Figure 2. ROC curve for in-hospital mortality within 30 days of diagnosis of a bacterial infection according to the neutrophil count> 7000/mm3 between Day 2 and Day 4. Supplementary Figure 3. ROC curve for in-hospital mortality within 30 days of diagnosis of a bacterial infection according to the C-reactive protein (CRP) > 100 mg/l between Day 2 and Day 4. [file 12877_2021_2515_MOESM1_ESM.docx]

**Supplementary Table 1.** Multivariate analysis of the factors associated with in-hospital mortality within 30 days of diagnosis of an infection

| **Variable** | **Deceased at Day 30,**  **n (%)** | **Crude HR**  **(95%CI%)** | **Adjusted HR**  **(95%CI)** | **P-value** |
| --- | --- | --- | --- | --- |
| **Age range** |  |  |  |  |
| *62 to 86 years old* | 7 (10.6) | 1 | 1 |  |
| *87 to 92 years old* | 15 (20.3) | 2.31 (0.94 to 5.67) | 3.09 (1.10 to 8.71) | 0.032 |
| *93 to 104 years old* | 14 (24.6) | 3.26 (1.30 to 8.18) | 6.18 (1.92 to 19.9) | 0.002 |
| **Sex** |  |  |  |  |
| *Women* | 16 (16.2) | 1 | 1 |  |
| *Men* | 20 (20.4) | 1.09 (0.56 to 2.11) | 1.54 (0.75 to 3.19) | 0.241 |
| **Charlson comorbidity index (+1)** |  | 1.06 (0.93 to 1.22) | 0.95 (0.82 to 1.11) | 0.535 |
| **Undernutrition** |  |  |  |  |
| *No* | 4 (11.1) | 1 | 1 |  |
| *Moderate* | 10 (14.3) | 1.28 (0.40 to 4.08) | 1.37 (0.40 to 4.75) | 0.618 |
| *Severe* | 22 (24.2) | 2.39 (0.82 to 6.95) | 2.91 (0.93 to 9.13) | 0.067 |
| **Infection type** |  |  |  |  |
| *Pulmonary* | 26 (23.2) | 1 | 1 |  |
| *Urinary* | 2 (4.7) | 0.24 (0.06 to 1.01) | 0.23 (0.05 to 1.03) | 0.036 |
| *Bacteraemia* | 5 (18.5) | 0.72 (0.28 to 1.89) | 0.52 (0.18 to 1.54) | 0.238 |
| *Combined or other* | 3 (20.0) | 0.82 (0.25 to 2.71) | 0.24 (0.05 to 1.15) | 0.074 |
| **GFR, n (%)** |  |  |  |  |
| *≥60 ml/min* | 16 (12.7) | 1 | 1 |  |
| *≥30 and <60 ml/min* | 9 (18.8) | 1.52 (0.67 to 3.44) | 1.82 (0.72 to 4.58) | 0.205 |
| *<30 ml/min* | 11 (30.6) | 4.46 (2.06 to 9.66) | 11.63 (4.08 to 33.1) | <0.001 |
| **Eosinophil count <100/mm^3^ between D2-D4** |  |  |  |  |
| *No* | 8 (7.0) | 1 | 1 |  |
| *Yes* | 28 (34.2) | 5.00 (2.28 to 11.0) | 8.90 (3.46 to 22.9) | <0.001 |

**Supplementary Table 2.** Performance of eosinophil count, C-reactive protein (CRP) and neutrophil count between Day 2 and Day 4 to predict in-hospital mortality within 30 days of diagnosis of a bacterial infection

| **Biomarker** | **Sensitivity** | **Specificity** | **Predictive Positive Value** | **Negative Predictive Value** |
| --- | --- | --- | --- | --- |
| **Eosinophil count** <100/mm^3^ | 77.8% | 66.5% | 34.2% | 93.0% |
| **Neutrophil count** >7000/mm^3^ | 77.8% | 57.1% | 28.9% | 92.0% |
| CRP >100 mg/L | 73.5% | 54.6% | 26.6% | 90.2% |


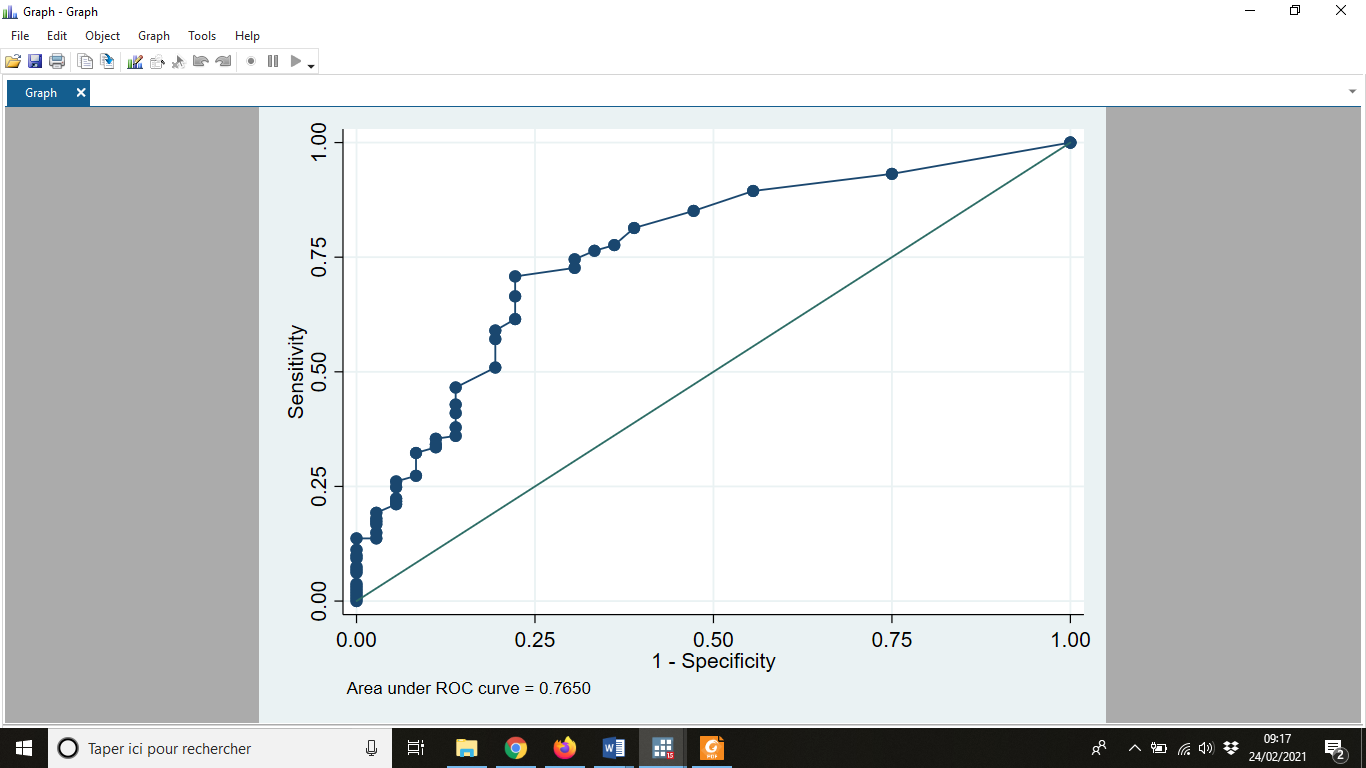


AUC=0.765, 95%CI [0.678 to 0.853]

**Supplementary Figure 1.** ROC curve for in-hospital mortality within 30 days of diagnosis of bacterial infection according to the eosinophil count>100/mm^3^ between Day 2 and Day 4. The classification variable was inverted in this analysis because higher eosinophil count is expected to predict survival.


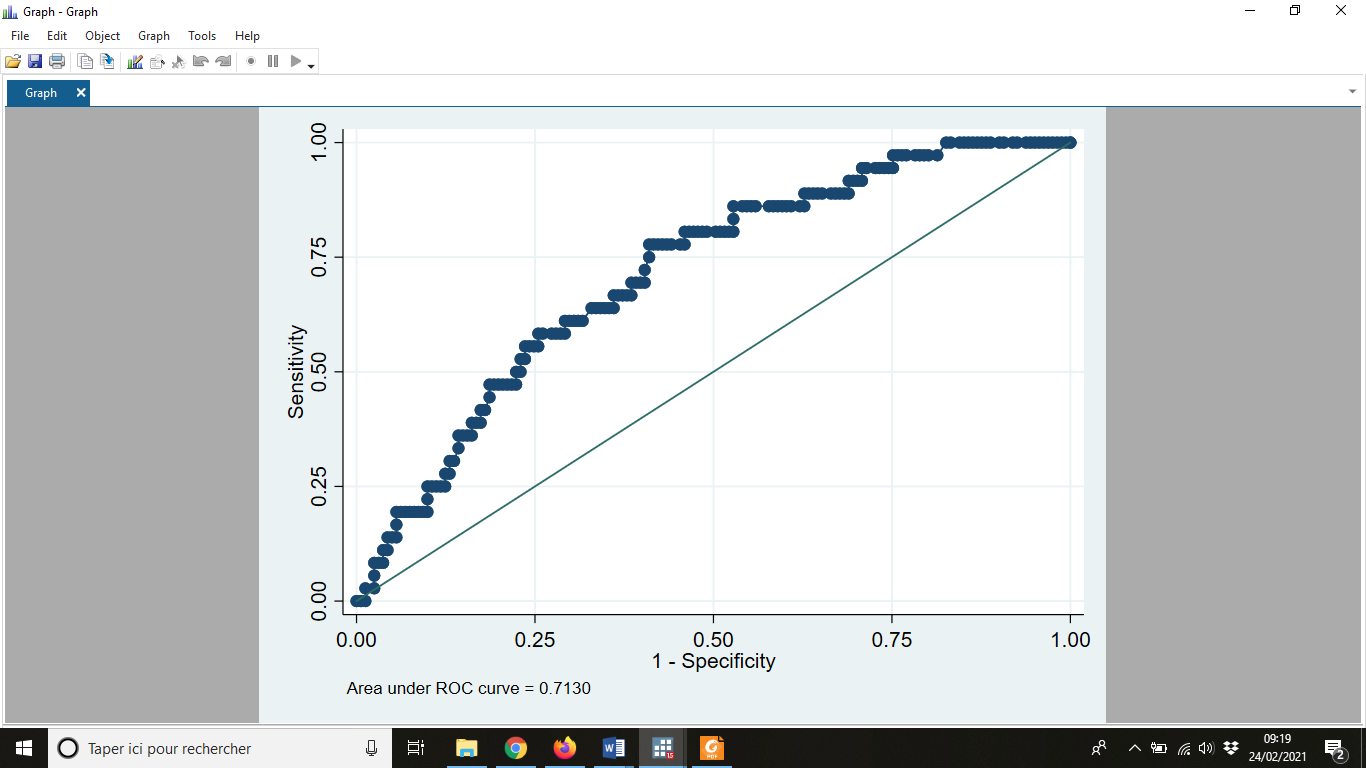


AUC=0.713, 95%CI [0.626 to 0.800]

**Supplementary Figure 2.** ROC curve for in-hospital mortality within 30 days of diagnosis of a bacterial infection according to the neutrophil count>7000/mm^3^ between Day 2 and Day 4


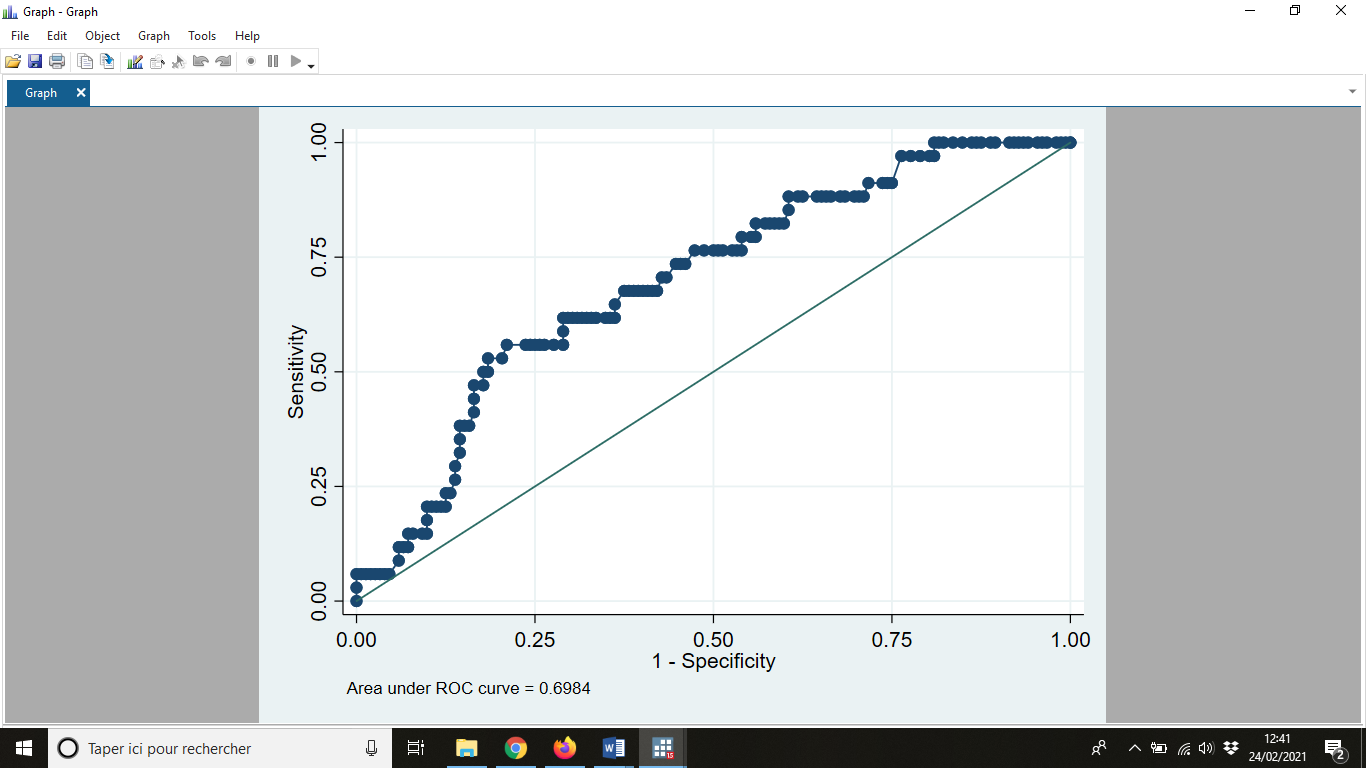


AUC=0.698, 95%CI [0.606 to 0.791]

**Supplementary Figure 3.** ROC curve for in-hospital mortality within 30 days of diagnosis of a bacterial infection according to the C-reactive protein (CRP)>100 mg/l between Day 2 and Day 4
